# Gastrointestinal health anti-diarrheal mixture relieves spleen deficiency-induced diarrhea through regulating gut microbiota

**DOI:** 10.1515/biol-2022-0964

**Published:** 2024-11-26

**Authors:** Zhengquan Wu, Liuyi Yao, Jun Guo, Zhong Xu, Zhengyan Wang

**Affiliations:** Department of Spleen and Stomach Diseases, Gansu Provincial Hospital of Traditional Chinese Medicine, No. 418, Guazhou Road, Qilihe District, Lanzhou, 730050, Gansu, China

**Keywords:** gastrointestinal health anti-diarrheal mixture, spleen deficiency, gut microbiota, diarrhea

## Abstract

This study evaluated the therapeutic efficacy of the gastrointestinal health anti-diarrheal mixture (GHAM) on diarrhea induced by spleen deficiency, focusing on its modulation of gut microbiota. Using specific pathogen-free Wistar rats, a spleen deficiency model was created through senna leaf gavage. Rats were divided into control, model, positive control, and GHAM treatment groups. After a 14-day treatment, fecal samples were analyzed via 16S rDNA sequencing to assess microbiota alterations. GHAM significantly mitigated diarrhea and enhanced food intake and fecal quality. It increased the abundance of beneficial bacteria, such as *Romboutsia* and *Clostridium_sensu_stricto_1*, and decreased the levels of diarrhea-associated bacteria, such as *Prevotellaceae* and *Bacillus*, thereby improving microbiota functionality. GHAM’s modulation of gut microbiota structure and function effectively alleviated spleen deficiency-induced diarrhea, positioning it as a potential natural herbal treatment for gastrointestinal ailments. This study lays the groundwork for further exploration of GHAM’s regulatory impact on gut health.

## 1 Introduction

In the traditional Chinese medicine (TCM) framework, the spleen is revered as a vital organ that encompasses a holistic integration of structure and function [1]. Its multifaceted roles in digestion, absorption, energy metabolism, and immune system regulation are fundamental to sustaining the body’s physiological balance [2]. The spleen’s influence on the body’s overall health is profound, and its deficiency can lead to a systemic weakening, particularly affecting the gastrointestinal system [2]. Diarrhea, when induced by spleen deficiency, emerges as a prevalent gastrointestinal disorder within TCM, presenting with symptoms such as loose stools, weight loss, and fatigue [3]. This condition sets itself apart from inflammatory bowel diseases and colitis, demanding a distinct approach to treatment and management.

The gut microbiota, a rich and intricate ecosystem of microorganisms, has been recognized for its significant contributions to intestinal health [4]. It plays a pivotal role in aiding digestion, synthesizing vitamins, and bolstering the immune system [4]. The composition and balance of the gut microbiota are closely intertwined with the spleen’s function, and disruptions in this balance have been observed in cases of spleen deficiency [5]. These disruptions can exacerbate diarrheal symptoms and contribute to the complexity of the condition.

Despite the clinical prevalence and impact of spleen deficiency-induced diarrhea, the search for effective treatments is ongoing. TCM offers a unique and holistic approach to addressing this condition, with herbal formulations such as the gastrointestinal health anti-diarrheal mixture (GHAM) demonstrating efficacy in clinical settings in China [6]. GHAM, a synergistic blend of traditional Chinese medicinal herbs, is believed to modulate the gut microbiota, thereby potentially alleviating the symptoms of diarrhea [7,8]. However, the precise mechanisms by which GHAM influences the gut microbiota and its overall therapeutic effects remain to be fully elucidated.

This study aims to address this gap in knowledge by conducting a comprehensive evaluation of the impact of GHAM on the gut microbiota in a rat model of spleen deficiency-induced diarrhea. Utilizing 16S rRNA gene sequencing, we will characterize the restructuring of the intestinal microbiota following GHAM treatment. The insights gained from this research could significantly contribute to the therapeutic potential of GHAM and enhance our understanding of TCM’s role in modulating gut health. Furthermore, this study will lay the groundwork for future explorations into the intricate relationship between the spleen, gut microbiota, and the treatment of gastrointestinal disorders.

## 2 Materials and methods

### 2.1 Preparation of GHAM

The GHAM formulation was obtained from the Scientific Research and Preparation Center of Gansu Provincial Hospital of Traditional Chinese Medicine. It is composed of a precise ratio of 11 medicinal herbs: *Agastache rugosa*, *Pueraria lobata*, citrus peel, *Coptis chinensis*, *Portulaca oleracea*, pomegranate peel, Chinese yam, *Saussurea costus*, *Atractylodes macrocephala*, *Glycyrrhiza glabra*, and *Poria cocos*, prepared in the following proportions: 2:2:2:1:2:2:3:1:3:1:3. The mixture was standardized to a concentration of 0.75 g of GHAM per milliliter of solution to ensure consistency in treatment administration.

### 2.2 *In vivo* experimental design

Specific pathogen-free Wistar rats, weighing 200 ± 20 g, were sourced from SiPeiFu Biotech (Beijing, SCXK 2019-0010). These animals were maintained under standardized conditions with a controlled temperature ranging from 20 to 26°C, relative humidity between 40 and 70%, and a 12 h light/dark cycle to simulate natural diurnal rhythms. Throughout the study, the rats were provided with a standard diet and water *ad libitum*. An acclimatization period of 1 week was observed prior to the commencement of experimental procedures to ensure their well-being and adaptability to the experimental environment. A total of 50 Wistar rats were evenly distributed into five experimental groups, each comprising ten individuals: a negative control (NC) group, a model group (M), a positive control group treated with medilac-vita (PC), and two GHAM treatment groups receiving low (ML) and high (MH) doses, respectively. With the exception of the NC group, all rats were subjected to daily gavage with a senna leaf solution at a dosage of 10 mL/kg body weight for a period of 30 consecutive days to induce the spleen deficiency condition. The PC group received 0.2 g/kg/day of medilac-vita, the ML group received 4.5 g/kg/day of GHAM, and the MH group received 18 g/kg/day of GHAM for 14 days. The NC group was administered an equivalent volume of physiological saline. Intestinal samples were collected at the end of the treatment period. The experimental design was conducted in strict accordance with the principles and guidelines recommended by the Chinese Association for Laboratory Animal Science and was approved by the Laboratory Animal Ethics Committee of Gansu University of Traditional Chinese Medicine. All procedures were performed under anesthesia with 0.6% pentobarbital sodium, and efforts were made to minimize animal suffering and reduce the number of animals used to obtain reliable data.


**Ethical approval:** The research related to animal use has been complied with all the relevant national regulations and institutional policies for the care and use of animals, and has been approved by the Laboratory Animal Ethics Committee of Gansu University of Traditional Chinese Medicine (Approval No. LL-202301300002).

### 2.3 Recording of loose feces and changes in food intake in rats

Food intake was measured in each group of rats before modeling and again at week 9 post-modeling. The criteria for successful replication of the diarrhea model with spleen deficiency were based on the Guiding Principles for Clinical Research of New Chinese Medicines. The rat model of spleen deficiency was evaluated using a combination of clinical evaluation criteria and quantitative indexes. A model was considered successful if the rat exhibited two or more symptoms of loose stools and decreased appetite, with a total score exceeding 5 points.

### 2.4 16S rDNA gene sequencing and analysis

Samples (200–500 mg) were weighed and pretreated. Total bacterial DNA from rat fecal samples was extracted using the magnetic bead method soil and fecal genomic DNA extraction kit (TianGen, China). DNA concentration and purity were assessed using a Qubit dsDNA HS Assay kit. DNA sample quality was controlled by 1% agarose gel electrophoresis. Amplification of the V3–V4 region of the 16S rRNA gene was performed using primers (forward: CCTAYGGGRBGCASGAG; reverse: GGACTACNNGGGGTATCTAAT). PCR products were purified by 2% agarose gel electrophoresis, and libraries were constructed using the NEB Next^®^ Ultra™ II FS DNA PCR-free Library Prep kit (New England Biolabs). The constructed libraries were quantified by Qubit and Q-PCR. After the library was qualified, PE250 upsequencing was performed using the NovaSeq 6000.

### 2.5 Statistical analysis

All experimental data are presented as mean ± standard deviation (*x* ± *s*). To ensure the robustness of our findings, a comprehensive suite of statistical tests was employed. Initially, a chi-square test of variance was conducted to assess normality and homogeneity of variance among groups. Subsequently, one-way analysis of variance (ANOVA) was utilized to detect significant inter-group differences.

Given the multiple comparisons, the Bonferroni correction was applied to adjust the significance threshold, reducing the likelihood of type I errors. The corrected alpha level (*α*′) was set to *α*/*n*, where *n* denotes the number of comparisons. This adjustment ensures that the family-wise error rate remains within acceptable limits.

For post-ANOVA pairwise comparisons, Tukey’s Honestly Significant Difference test was used, allowing for the determination of groups which significantly differ while controlling the overall type I error rate.

In addition to parametric tests, non-parametric analyses were conducted for data not meeting normality assumptions. The Kruskal–Wallis test served as an alternative to ANOVA, with the Mann–Whitney *U*-test applied for pairwise comparisons of non-normally distributed data.

Statistical significance was set at *P* < 0.05. All analyses were performed using SPSS version 22.0, facilitating the management and interpretation of complex datasets.

## 3 Results

### 3.1 Successful construction of a rat model of spleen deficiency-induced diarrhea

**Figure 1:**
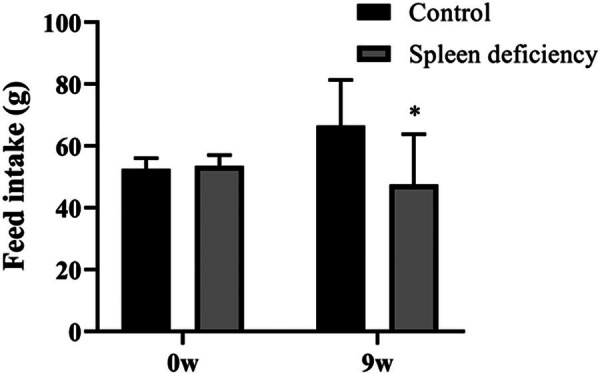
Successful construction of a rat model of spleen deficiency-induced diarrhea by continuous gavage of senna solution. Changes in food intake of rats before and after modeling. *Represents a significant difference compared with the control group.

Following 30 days of continuous senna gavage, rats exhibited characteristic loose stools. A significant reduction in food intake was observed at the ninth week post-modeling (Figure 1).

### 3.2 Venn diagram of intestinal flora species abundance profiles and distribution of characteristic sequences

**Figure 2:**
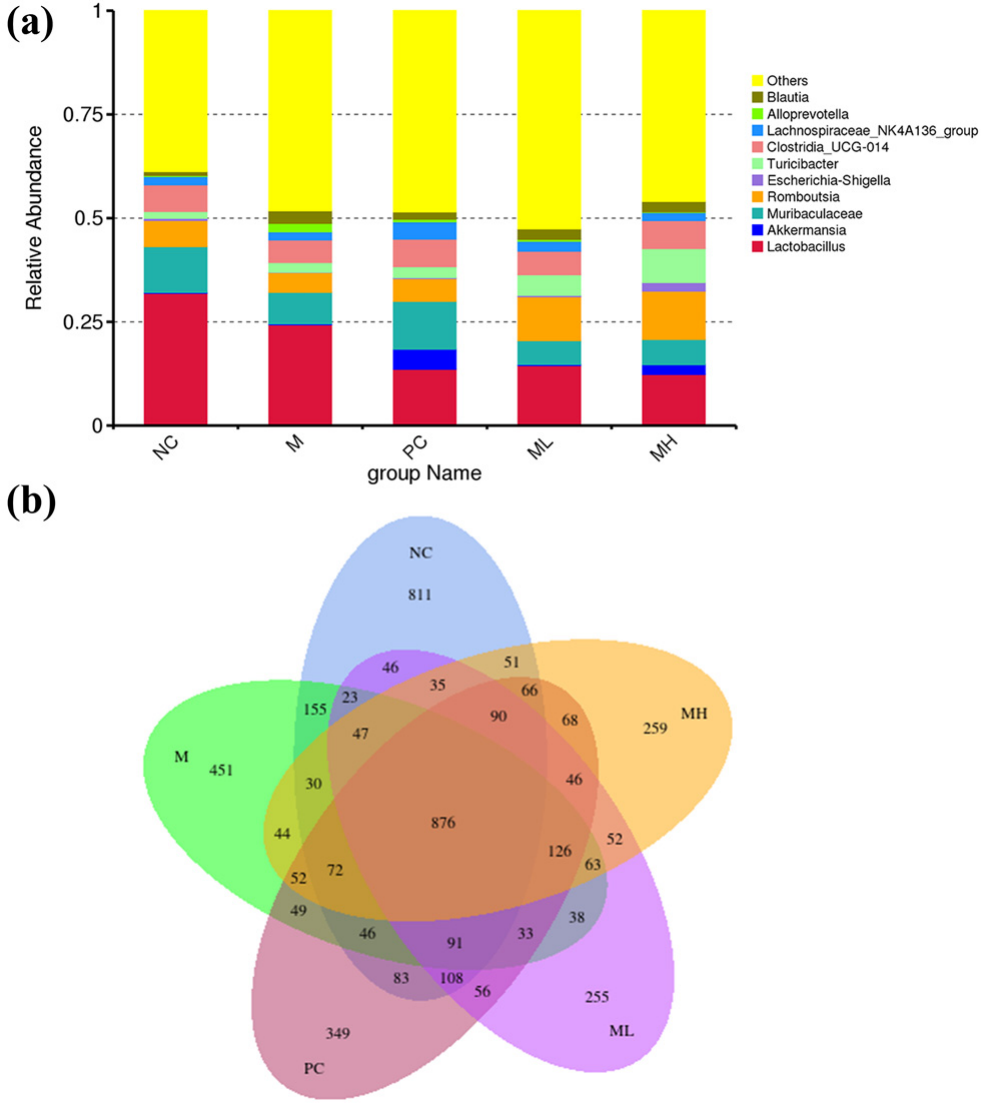
Plot of the abundance of genera and Venn diagram of the distribution of characteristic sequences in each group. (a) Abundance of groups at genus taxonomic level. (b) Venn diagram of the distribution of the characteristic sequences for each group. Note: Each circle represents a group of samples, the number of overlapping circles represents the number of feature sequences common to the sample group, the numbers without overlapping parts represent sequences unique to the sample group.

The chart shows the top ten genera in each group at the genus classification level, with the most abundant genera being *Blutia*, *Alloprevotella*, *Lachnospiraceae*, *Clostridia*, *Turicibacter*, *Escherichia-Shigella*, *Romboutsia*, *Muribaculaceae*, *AKKermansia*, and *Lactobacillus* (Figure 2a). The NC, M, PC, ML, and MH groups contained 876 shared sequences. The numbers of unique sequences in the NC, M, PC, ML, and MH groups were 811, 451, 349, 255, and 259, respectively (Figure 2b).

### 3.3 Alpha index of diversity and beta index of diversity

**Figure 3:**
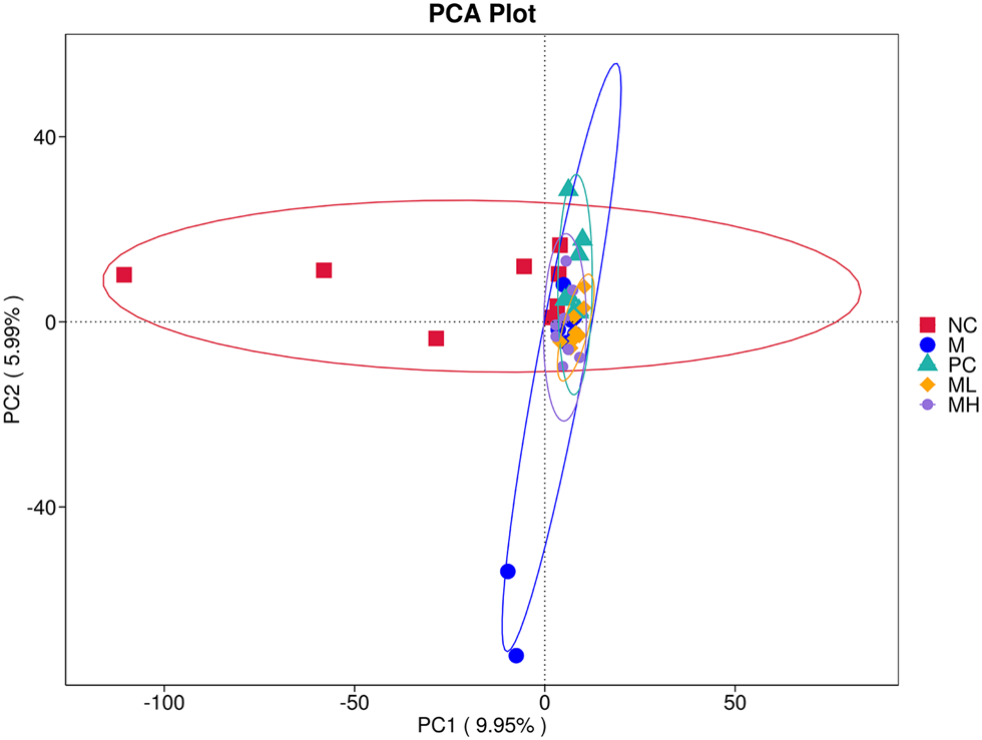
Alpha diversity table and PCA plot of beta diversity. Note: In a PCA plot, the closer the distance between groups, the more similar the community composition of the samples.

The Alpha diversity index of each group is shown in Table 1, which is closer and not significantly different between the groups, indicating that the diversity and homogeneity of the colonies in each group were closer. The NC and model groups had the most pronounced differences in community composition, and the samples were more dispersed within these two groups. The ML and MH groups had more concentrated samples within the groups and were more similar in terms of community composition (Figure 3).

**Table 1:** Alpha index of diversity

Sample_Name	Chao1	Dominance	Goods_coverage	Observed_features	Pielou_e	Shannon	Simpson
NC	2649.06	0.029	1	2,630	0.685	7.779	0.971
M	2209.611	0.02	1	2,196	0.694	7.704	0.98
PC	2222.168	0.011	1	2,211	0.75	8.338	0.989
ML	2013.088	0.023	1	1,985	0.698	7.643	0.977
MH	1993.405	0.021	1	1,977	0.695	7.613	0.979

Note: Chao1: estimating the total number of species contained in a community sample; Dominance: the probability that two sequences, taken at random, are from the same sample; Goods_coverage: coverage of sequencing; Observed_features: number of species visually observed; Pielou _e: homogeneity index; Shannon: total number of classifications in the sample and their percentage; Simpson: characterization of the diversity and evenness of species distributions within communities.

### 3.4 Differences in changes in the content of intestinal bacterial genera among groups of rats

**Figure 4:**
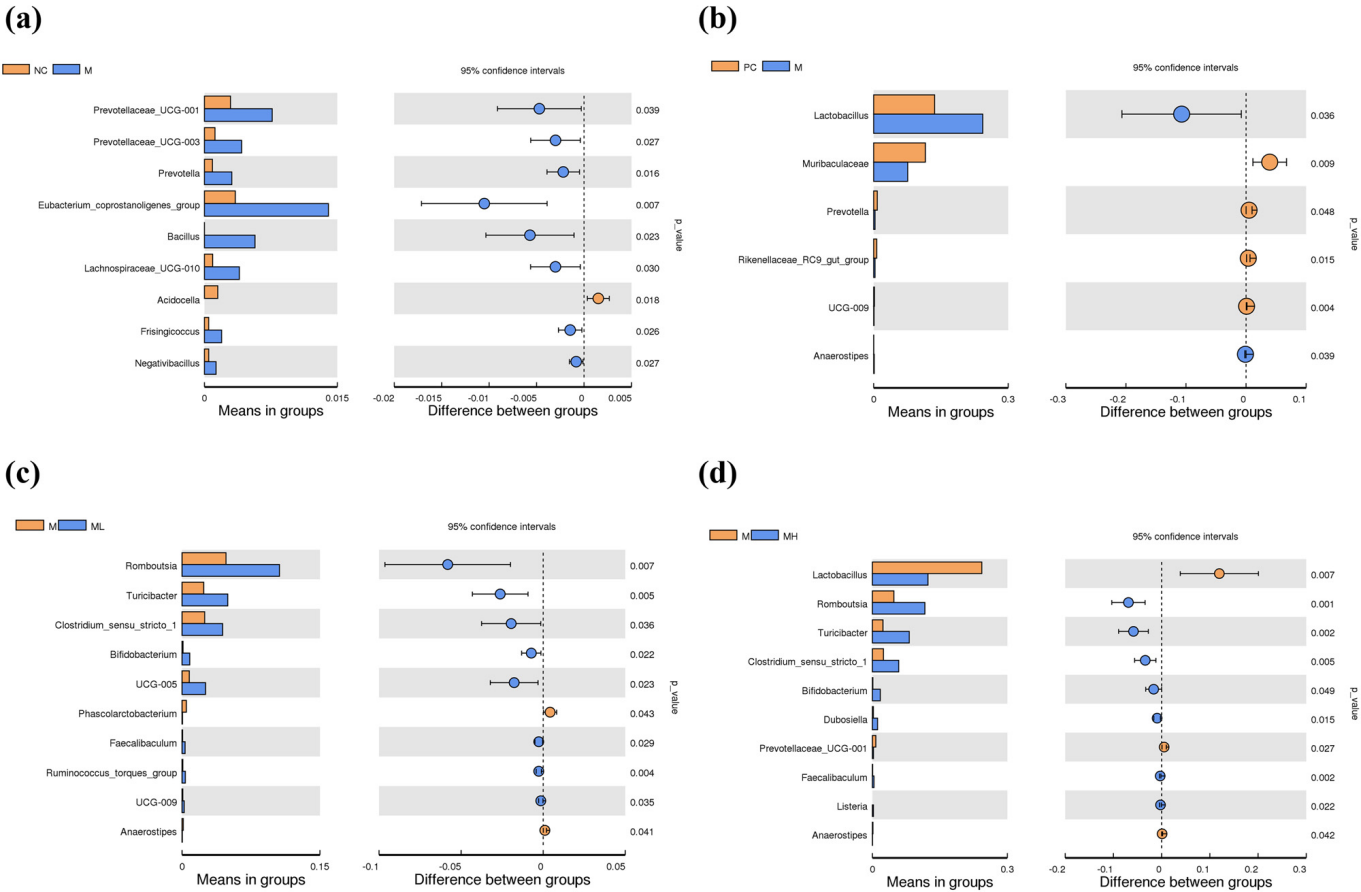
Plot of differences in changes in genus content. (a) Bacterial genus plot of the NC group compared to the M group. (b) Bacterial genus plot of the PC group compared to the M group. (c) Bacterial genus plot of the M group compared to the ML group. (d) Bacterial genus plot of the M group compared to the MH group.

Comparisons were made at the genus level using the *t*-test ANOVA. In the NC group, compared with the M group, *Prevotellaceae*, *Prevotella*, *Eubacterium_coprostanoligenes_group*, *Lachnospiraceae_UGG-010*, *Bacillus*, *Frisingicoccus,* and *Negativibacillus* were significantly downregulated, while *Acidocella* was significantly upregulated (Figure 4a). In the PC group, compared with the M group, *Lactobacillus* and *Anaerostipes* were significantly downregulated, whereas *Muribaculaceae*, *Prevotella*, *Rikenellaceae_RC9_gut_group*, and *UGG_009* were significantly upregulated (Figure 4b). In the M group compared to the ML group, *Romboutsia*, *Turicibacter*, *Clostridium_sensu_stricto_1*, *Bifidobacterium*, *UGG_005*, *Faecalibaculum*, *Ruminococcus_torques_group*, and *UGG_009* were significantly downregulated, and *Phascolarctobacterium* and *Anaerostipes* were significantly upregulated (Figure 4c). In the M group compared with the MH group, *Romboutsia*, *Turicibacter*, *Clostridium_sensu_stricto_1*, *Bifidobacterium*, *Dubosiella*, *Faecalibaculum*, and *Listeria* were significantly downregulated, and *Lactobacillus*, *Prevotellaceae_UGG-001*, and *Anaerostipes* were significantly upregulated (Figure 4d).

### 3.5 Functional prediction and heat map of rat intestinal flora

**Figure 5:**
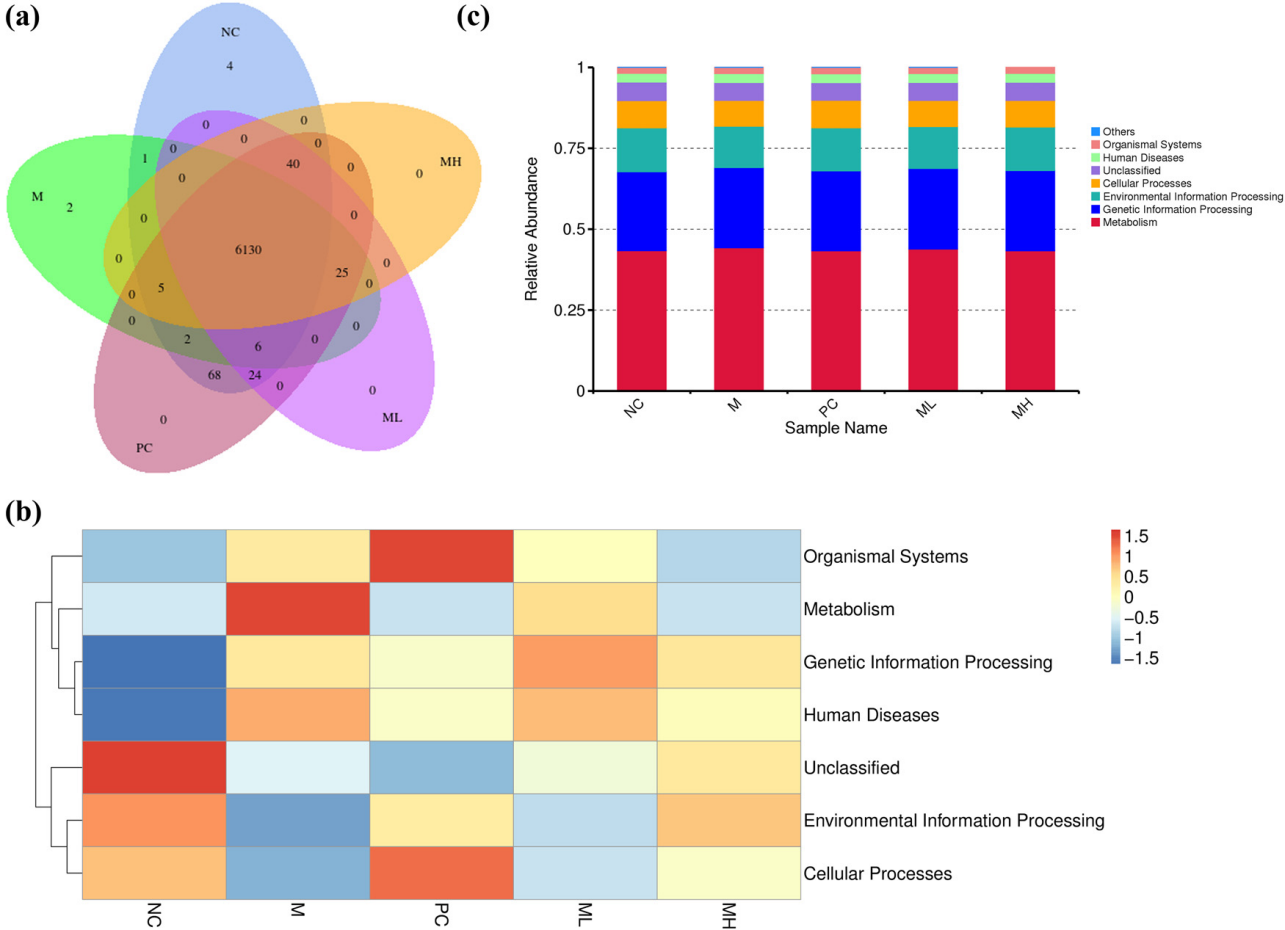
Functional prediction and heat map of rat intestinal flora. (a) Venn diagram of functional prediction distribution. (b) Heat plot of functional prediction. (c) Abundance plot of functional prediction.

According to the results of the Venn diagram, the number of the same functions in the NC, M, PC, ML, and MH groups was 6,130. The numbers of unique functions of the NC, M, PC, ML, and MH groups were 4, 2, 0, 0, and 0, respectively (Figure 5a).

Using the Tax4Fun function, bacteria in the NC, M, PC, ML, and MH groups were predicted to play roles in organismal systems, human diseases, cellular processes, environmental information processing, genetic information processing, and metabolic pathways (Figure 5b).

Compared to the PC and NC groups, the function of the bacteria in the M group was weakened in cellular processes, environmental information processing, and increased in metabolism, genetic information processing, and human diseases. Compared to the M group, the function of the bacteria in the ML group was weakened in metabolism and strengthened in genetic information processing, cellular processes, and environmental information processing. Compared with the M group, the function of the bacteria in the MH group was weakened in organismal systems, metabolism, and human diseases and strengthened in genetic information processing and cellular processes (Figure 5c).

## 4 Discussion

GHAM effectively tonifies the spleen and boosts qi. Qi facilitates and invigorates the spleen’s physiological functions, reflecting its dynamic flow, known as spleen qi circulation. Diminished spleen qi impairs the spleen’s transportation and transformation abilities, consequently disrupting food digestion and reducing the assimilation and distribution of vital nutrients throughout the body. This condition leads to a loss of appetite, bloating, loose bowel movements, and emaciation.

The GHAM formulation is a refined and synergistic blend of Huo-xiang-zheng-qi powder [9], Ge-gen-qin-lian decoction [10], and Sijunzi decoction [11], incorporating 11 types of traditional Chinese medicinal herbs. *A. rugosa* [12] serves to regulate gastrointestinal function, purify the internal environment, and reduce dampness. *P. lobata* [13] exhibits heat-clearing and diuretic properties, enhancing Yang energy, arresting diarrhea, and aiding in the production of body fluids. Citrus peel [14] regulates qi and restores splenic function. *C. chinensis* [15] can dry dampness, clear heat, extinguish fire, and detoxify. *P. oleracea* [16] can clear heat, detoxify and lower blood pressure, and treat diarrhea. Pomegranate peel [17] can stop diarrhea by reducing excessive activity in the intestines. Chinese yam [18] can strengthen the spleen and intestines, contributing to a healthy body. *S. costus* [19] can strengthen the spleen and promote qi. *A. macrocephala* [20], *G. glabra* [21], and *P. cocos* [22] have analgesic effects. Overall, this formula regulates the stomach and intestines, adjusts inner dampness, regulates qi and blood circulation, and relieves symptoms of excessive intestinal activity.

In this study, we successfully constructed a rat model of spleen deficiency-induced diarrhea by continuous gavage of senna, and the rats showed a significant reduction in food intake and diarrhea phenotype. We can also conclude the success of the diarrhea model from the results of the comparison at the genus level of the flora in the M and NC groups. In the M group, there was a notable increase in the levels of *Prevotellaceae* [23], *Prevotella* [24], *Bacillus* [25], and *Negativibacillus* [26], all of which are implicated in the pathophysiology of diarrhea. Relative to the M group, the ML and MH groups exhibited elevated levels of *Romboutsia*, *Clostridium_sensu_stricto_1*, and *Turicibacter*, indicating a positive modulation of the gut microbiota. *Romboutsia* [27] and *Clostridium_sensu_stricto_1* [28] are beneficial bacteria. *Turicibacter* is a key gut microbe that suppresses persistent systemic inflammation [29]. The ML and MH groups demonstrated a moderated metabolic function, coupled with an enhancement in cellular processes and genetic information processing relative to the M group. The results indicate that GHAM treatment partially reinstated the functionality of the intestinal bacteria, approximating them to their normal state.

Furthermore, GHAM treatment reinforces the metabolic capabilities and genetic information processing of the gut microbiota. Relative to the model group, the ML and MH groups exhibited a dampened metabolic activity, counterbalanced by an upregulation in genetic information processing and cellular processes. This suggests that GHAM may improve gut health by promoting the growth of beneficial bacteria and enhancing the beneficial functions of the gut microbiota.

The results of this study indicate that GHAM has shown positive therapeutic effects in a diarrhea rat model induced by spleen deficiency. By regulating the structure and function of the gut microbiota, GHAM significantly alleviated diarrhea symptoms and improved food intake and fecal quality. In particular, GHAM increased the abundance of beneficial bacteria such as *Romboutsia* and *Clostridium_sensu_stricto_1*, while reducing the levels of bacteria associated with diarrhea, such as *Prevotellaceae* and *Bacillus*.

However, we must cautiously point out that these conclusions are based on preliminary research results from animal models. Although these findings provide some scientific basis for GHAM as a potential treatment for gastrointestinal diseases, they do not mean that GHAM can be directly applied to human clinical treatment. Before applying GHAM as a therapeutic method in humans, more research is needed, including further animal studies and human clinical trials, to verify its safety, efficacy, and mechanism of action.

In addition, our research results also suggest that the regulatory effect of GHAM on the gut microbiota may be related to its therapeutic effects. Future research can further explore the specific impact of GHAM on the gut microbiota and how these impacts are associated with improving intestinal health and alleviating diarrhea symptoms.

In summary, this study provides evidence of the potential therapeutic effect of GHAM on diarrhea caused by spleen deficiency and lays the foundation for future research. However, further research is needed to verify these preliminary results and fully assess the potential of GHAM as a treatment method for gastrointestinal diseases.
